# Curriculum and assessment tool for less invasive surfactant administration: an international Delphi consensus study

**DOI:** 10.1038/s41390-023-02621-2

**Published:** 2023-05-04

**Authors:** Niklas Breindahl, Martin G. Tolsgaard, Tine B. Henriksen, Charles C. Roehr, Tomasz Szczapa, Luigi Gagliardi, Maximo Vento, Ragnhild Støen, Kajsa Bohlin, Anton H. van Kaam, Daniel Klotz, Xavier Durrmeyer, Tongyan Han, Anup C. Katheria, Peter A. Dargaville, Lise Aunsholt

**Affiliations:** 1 Department of Neonatal and Pediatric Intensive Care, Copenhagen University Hospital, Rigshospitalet, Copenhagen, Denmark; 2 Copenhagen Academy for Medical Education and Simulation (CAMES), Copenhagen University Hospital, Rigshospitalet, Copenhagen, Denmark; 3 Prehospital Center Region Zealand, Næstved, Denmark; 4 Department of Clinical Medicine, University of Copenhagen, Copenhagen, Denmark; 5 Department of Obstetrics, Copenhagen University Hospital Rigshospitalet, Copenhagen, Denmark; 6 Department of Paediatrics (Intensive Care Neonatology), Aarhus University Hospital, Aarhus, Denmark; 7 Perinatal Research Unit, Clinical Institute, Aarhus University, Aarhus, Denmark; 8 Newborn Services, Southmead Hospital, North Bristol NHS Trust Bristol, Bristol, UK; 9 Nuffield Department of Population Health, National Perinatal Epidemiology Unit, Medical Sciences Division, University of Oxford, Oxford, UK; 10 2nd Department of Neonatology, Neonatal Biophysical Monitoring and Cardiopulmonary Therapies Research Unit, Poznan University of Medical Sciences, Poznan, Poland; 11 Division of Neonatology and Pediatrics, Ospedale Versilia, Viareggio, Azienda USL Toscana Nord Ovest, Pisa, Italy; 12 Division of Neonatology, University and Polytechnic Hospital La Fe (HULAFE) and Health Research Institute (IISLAFE), Valencia, Spain; 13 Department of Neonatology, St. Olavs Hospital, Trondheim University Hospital, Trondheim, Norway; 14 Department of Clinical and Molecular Medicine, Norwegian University of Science and Technology, Trondheim, Norway; 15 Department of Neonatology, Karolinska University Hospital and Karolinska Institutet, Stockholm, Sweden; 16 Department of Neonatology, Emma Children’s Hospital Amsterdam UMC, University of Amsterdam, Amsterdam, The Netherlands; 17 Center for Pediatrics, Division of Neonatology and Pediatric Intensive Care Medicine, Medical Center - University of Freiburg, Faculty of Medicine, University of Freiburg, Freiburg, Germany; 18 Department of Neonatal Intensive Care and Neonatology, Centre Hospitalier Intercommunal de Créteil, Université Paris Est Créteil, Créteil, France; 19 GRC CARMAS, IMRB, Université Paris Est Créteil, Faculté de Santé de Créteil, Créteil, France; 20 Department of Pediatrics, Peking University Third Hospital, Beijing, China; 21 Neonatal Research Institute, Sharp Mary Birch Hospital for Women & Newborns, San Diego, CA 92123 USA; 22 Department of Paediatrics, Royal Hobart Hospital, Menzies Institute for Medical Research, University of Tasmania, Hobart, Australia; 23 Department of Veterinary and Animal Science, University of Copenhagen, Copenhagen, Denmark

## Abstract

**Background:**

Training and assessment of operator competence for the less invasive surfactant administration (LISA) procedure vary. This study aimed to obtain international expert consensus on LISA training (LISA curriculum (LISA-CUR)) and assessment (LISA assessment tool (LISA-AT)).

**Methods:**

From February to July 2022, an international three-round Delphi process gathered opinions from LISA experts (researchers, curriculum developers, and clinical educators) on a list of items to be included in a LISA-CUR and LISA-AT (Round 1). The experts rated the importance of each item (Round 2). Items supported by more than 80% consensus were included. All experts were asked to approve or reject the final LISA-CUR and LISA-AT (Round 3).

**Results:**

A total of 153 experts from 14 countries participated in Round 1, and the response rate for Rounds 2 and 3 was >80%. Round 1 identified 44 items for LISA-CUR and 22 for LISA-AT. Round 2 excluded 15 items for the LISA-CUR and 7 items for the LISA-AT. Round 3 resulted in a strong consensus (99–100%) for the final 29 items for the LISA-CUR and 15 items for the LISA-AT.

**Conclusions:**

This Delphi process established an international consensus on a training curriculum and content evidence for the assessment of LISA competence.

**Impact:**

- This international consensus-based expert statement provides content on a curriculum for the less invasive surfactant administration procedure (LISA-CUR) that may be partnered with existing evidence-based strategies to optimize and standardize LISA training in the future.
- This international consensus-based expert statement also provides content on an assessment tool for the LISA procedure (LISA-AT) that can help to evaluate competence in LISA operators. The proposed LISA-AT enables standardized, continuous feedback and assessment until achieving proficiency.

## Introduction

Less invasive surfactant administration (LISA) is a technique to administer surfactant to preterm infants with respiratory distress syndrome (RDS). LISA is performed by placing a thin catheter below the vocal cords of spontaneously breathing infants on non-invasive respiratory support.^1–4^ Since the 2016 update of the European Consensus Guidelines on Management of Respiratory Distress Syndrome,^5^ LISA has been suggested as the preferred method of surfactant administration in spontaneously breathing premature infants and is now widely used in neonatal intensive care units (NICUs) globally.^6–9^ However, the risks of airflow obstruction, apnea, desaturations, bradycardia, misplacement of the catheter, pain and discomfort persist.^10^ Video laryngoscopy may reduce some of these adverse events.^11,12^ Effective and safe performance of LISA requires an experienced operator and supporting clinical team.^13^ However, operator and team’s experience with LISA may be limited due to the relatively small number of infants eligible for LISA per clinician. Standardized simulation-based LISA education, including use of video laryngoscopy, has been recommended to improve LISA success rates.^14–17^ Nonetheless, to date, there is a lack of consensus on clinical practice and training required for gaining LISA proficiency.^18^ Therefore, clinicians would benefit from an internationally consented curriculum, based on sound clinical evidence and expert’s experience, that will serve as a template for LISA accreditation. Curriculum development should follow a structured approach starting with problem identification and a general needs assessment.^19,20^ Further, to support skills development, methods for assessment of LISA competence are needed to ensure high and consistent performance and to enable future mastery learning of the LISA procedure, where trainees practice with feedback until they achieve a predefined mastery learning level.^21,22^ This study aimed to provide international expert consensus on (1) the training curriculum for LISA operators and (2) what to include in the assessment of LISA competence.

## Methods

### Study design

**Fig. 1:**
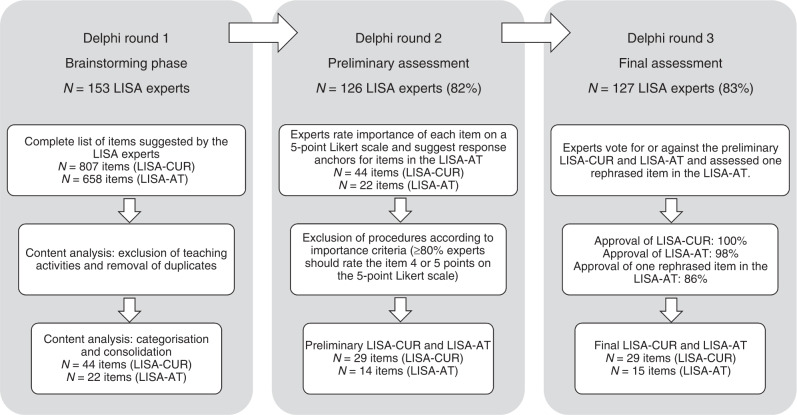
Overview of the three-round Delphi process from February to July 2022. All experts who participated in the first round were invited to participate in the following rounds. LISA less invasive surfactant administration, LISA-CUR LISA curriculum, LISA-AT LISA assessment tool.

From February to July 2022, we conducted a three-round iterative Delphi process using online survey questionnaires to collect information and establish consensus regarding the content to be included in (1) a LISA curriculum (LISA-CUR) and (2) a LISA assessment tool (LISA-AT) for the assessment of LISA competence. The Delphi process is a systematic, group facilitation technique using structured and semi-structured questionnaires to collect and organize the opinions from experts in the field until consensus is reached.^23^ The survey questionnaires for Delphi rounds 1–3 are available from the corresponding author. We defined consensus in rounds 2 and 3 as an agreement of ≥80%. Figure 1 presents an overview of the process.

### Data collection

We used the Research Electronic Data Capture (REDCap) system for data collection. Chinese experts could not access REDCap from mainland China and submitted their replies by e-mail. This information was subsequently entered into REDCap by the principal investigator (N.B.).

### Sample characteristics

The LISA experts were identified based on their involvement in research, curriculum development, and clinical education in the LISA procedure.

Invitations to participate were sent via e-mail by the steering committee members using their professional networks. The initial invitation to participate was promoted by the European Society for Paediatric Research (ESPR) via its website, on social media, and in its newsletter. The invitation included a link to the online consent form and the survey questionnaire for round one. Participation was voluntary. All experts who participated in the first round were invited to participate in the following rounds.

### Survey administration

A Delphi steering committee was established to facilitate and manage all the steps in the Delphi process, including recruitment of experts and data analyses. The steering committee included an international team of 16 members with experience in neonatal intensive care or postgraduate medical education. All steering committee members except N.B. and M.G.T. were invited to participate in the survey due to their roles as international experts on the LISA procedure. The survey was developed by N.B. and piloted by the steering committee before the data collection in each round.

### Delphi round 1

The first round was a brainstorming phase where experts replied to the following statements: (1) “*List, in free text, the specific knowledge and skills that you believe a newly certified LISA operator needs to have and should be included in a curriculum for LISA training*”, and (2) “*List, in free text, all relevant aspects of the LISA procedure to be included in an assessment tool to evaluate competence/certification of a LISA operator*.”

Experts provided baseline information, including sex, age, specialty, and years of experience with the LISA procedure and answered questions concerning local LISA procedure practice and use of training equipment.

Data were gathered by N.B. and processed by the steering committee. Duplicates were removed. Teaching activities, learning-teaching strategies, and teaching methods were omitted as they were already described in other publications.^15^ The remaining items were analyzed using content analysis^24^ to identify repeated categories of procedures and various textual expressions for each unique item to consolidate the lists for the LISA-CUR and the LISA-AT used in round 2. Due to variations in phraseology in round 1, the steering committee was allowed to adjust the wording but not the content of each suggested item. Decisions were based on 100% agreement within the steering committee.

### Delphi round 2

In this round, items generated in the *Delphi round 1* were assessed for suitability for the LISA-CUR and the LISA-AT. The selection was based on importance by using a five-point Likert scale; (1) Not important at all, (2) Less important, (3) Neutral, (4) Important, (5) Very important. The experts replied to the following statements individually for each item: (1) “*How important are the following items when learning how to perform the LISA procedure?*” and (2) “*How important are the following items when assessing the skill of an operator performing the LISA procedure?*”

Items rated as important (4) or very important (5) by 80% or more of the experts were included in the preliminary LISA-CUR and LISA-AT that were sent back to the experts for final review and comments in *Delphi round 3*.

### Delphi round 3

The experts were informed about the distribution of scores, the excluded items, and selected comments from other experts in round 2. Subsequently, they were asked to approve or reject and comment on the preliminary LISA-CUR and LISA-AT.

### Ethical considerations

Ethical approval was waived by the Committee on Health Research Ethics in the Capital Region of Denmark (Journal number: 21051793). Experts were informed about the aims of this study, the importance of participation, and the detailed tasks in each step of the Delphi process. All experts provided electronic informed consent prior to study participation. The datasets were de-identified prior to analyses. All information was handled confidentially. Data management and processing were approved (ID-number: P-2022-11). This study is reported according to the Consensus-Based Checklist for Reporting of Survey Studies (CROSS)^25^ (Online Supplement, Appendix A).

### Statistical analysis

Categorical data from *Delphi round 1* are presented as frequencies (counts and percentages) and continuous data as medians with interquartile ranges [IQRs] and range as appropriate. Statistical analyses were performed using RStudio version 1.2.5001. There were no changes to study design after this study commenced. There was no imputation of missing data. The required response rate for each round was ≥70% to minimize non-response error.

## Results

### Expert demographics

One hundred fifty-three experts agreed to participate in this study representing 14 countries worldwide (Table 1). Experts were involved in research (48%), curriculum development (56%), and clinical education (87%) regarding the LISA procedure, and the median [IQR] time of involvement in the LISA procedure was 5 [4–8] years. Most were board-certified neonatologists (97%), 16% in pediatrics, 1% in anesthesia, and 1% in epidemiology. More than half the experts (54%) worked at hospitals with more than 10 individual LISA operators. According to the experts, 37% of LISA operators in their respective units performed less than one procedure per month, and 52% performed 1–5 procedures per month (Table 1).

**Table 1:** Experts’ baseline information.

Category	LISA experts (*n* = 153)
Sex (male), *n* (%)	78 (51%)
Age (years), median [IQR], range	51 [45–57], 32–70
Areas of experience, *n* (%)^a^	
Research	73 (48%)
Curriculum development	85 (56%)
Clinical education	133 (87%)
Years of LISA experience, median [IQR]	5 [4–8]
Specialty, *n* (%)^a^	
Neonatology	149 (97%)
Pediatrics	25 (16%)
Anesthesia	1 (1%)
Other (epidemiology)	1 (1%)
Number of LISA operators per hospital, *n* (%)	
<4	2 (1%)
4	6 (4%)
5	9 (6%)
6	16 (10%)
7	6 (4%)
8	18 (12%)
9	4 (3%)
10	9 (6%)
>10	82 (54%)
Monthly incidence of LISA procedures per operator, *n* (%)	
<1	56 (37%)
1–5	79 (52%)
6–10	10 (7%)
11–15	3 (2%)
16–20	3 (2%)
21–30	0 (0%)
>30	1 (1%)
Nationality, *n* (%)	
Denmark	23 (15%)
Spain	22 (14%)
Norway	13 (8%)
China	11 (7%)
Germany	11 (7%)
Italy	11 (7%)
Netherlands	11 (7%)
France	10 (7%)
Poland	9 (6%)
United Kingdom	9 (6%)
Sweden	8 (5%)
Australia	7 (5%)
United States of America	7 (5%)
Austria	1 (1%)
Number of items suggested for the LISA-CUR, median [IQR]	5 [3–7]
Number of items suggested for the LISA-AT, median [IQR]	4 [3–6]

LISA experts’ baseline information in round 1 (*n* = 153).

*LISA* less invasive surfactant administration, *LISA-CUR* LISA curriculum, *LISA-AT* LISA assessment tool.

^a^More than one option could be chosen.

### The LISA procedure

Based on the experts’ replies, median gestational age of infants considered eligible for treatment with the LISA procedure was 28 weeks (IQR 24–32 weeks, range 22–42 weeks) (Table 2). nCPAP was the most frequently reported mode of respiratory support used during LISA (84%), followed by NIPPV (46%) and HFNC (9%).

**Table 2:** Experts’ report of therapies applied during LISA.

Category	Results
Report of gestational age for eligible infants, median [IQR], range	28 [24–32], 22–42
Report of analgesic/sedative pharmacological treatment, *n* (%)^a^	
Fentanyl	75 (49%)
Propofol	28 (18%)
Ketamine	17 (11%)
Lidocain spray	2 (1%)
Midazolam	7 (5%)
Phenobarbital	2 (1%)
Morphine	7 (5%)
Sufentanil	1 (1%)
No use of analgesics/sedatives	63 (41%)
Report of non-pharmacological treatment, *n* (%)^a^	
Swaddling/wrapping	89 (58%)
Sucrose	73 (48%)
Tucking	70 (46%)
Skin-to-skin care	23 (15%)
Environment (light and noise reduction)	19 (12%)
Containment/holding	15 (10%)
Positioning	10 (7%)
Non-nutritive suctioning	6 (4%)
NIDCAP principles	3 (2%)
Human milk	3 (2%)
No use of non-pharmacological treatment	6 (4%)
Report of surfactant used, *n* (%)	
Curosurf/Poractant alfa/Porcine surfactant	125 (94%)
Calsurf/Bovine surfactant	8 (6%)
Report of pre-procedure pharmacological treatment, *n* (%)^a^	
Caffeine	138 (90%)
Atropine	35 (23%)
Paracetamol	2 (1%)
No use of pre-procedure pharmacological treatment	11 (7%)
Report of post-procedure pharmacological treatment, *n* (%)^a^	
Naloxone	36 (24%)
Caffeine	7 (5%)
Theophylline	1 (1%)
Paracetamol	1 (1%)
Antibiotics	1 (1%)
Atropine	1 (1%)
Doxapram	1 (1%)
No use of post-procedure pharmacological treatment	106 (69%)
Report of respiratory support during LISA, *n* (%)^a^	
nCPAP	129 (84%)
NIPPV	71 (46%)
HFNC	14 (9%)
Other respiratory support during LISA	9 (6%)

Therapies applied during LISA according to the 153 experts.

*HFNC* high-flow nasal cannula, *IQR* interquartile range, *LISA* less invasive surfactant administration, *nCPAP* nasal continuous positive airway pressure, *NIDCAP* Newborn Individualized Developmental Care and Assessment Program, *NIPPV* nasal intermittent positive pressure ventilation.

^a^More than one option could be chosen.

Forty-one per cent of experts reported no use of pre-procedure sedatives or analgesics, whereas 49% indicated the use of fentanyl as pre-procedure treatment (Table 2). Use of non-pharmacological measures was reported by 96% of the experts: 58% used swaddling/wrapping, 48% used sucrose, 46% used tucking and other interventions like skin-to-skin care, light and noise reduction, positioning, and pre-procedure non-nutritive suctioning.

### Main findings

The final LISA-CUR and the LISA-AT, including response anchors for guided assessments, are available in Tables 3 and 4, respectively.

**Table 3:** The final approved LISA curriculum (LISA-CUR).

Indications and contraindications
Knowledge about the indications and limitations of the LISA procedure
Knowledge about the effect of surfactant on pulmonary function
Knowledge about how to assess the severity of RDS according to local protocol
Knowledge on the most recent relevant guidelines on RDS
Complications
Knowledge about the incidence, signs, and management of complications during the LISA procedure
Familiarity with the equipment
Familiar with equipment required for the LISA procedure
Practical skills in inserting and positioning the catheter in the trachea at the correct depth
Practical skills in maintaining the correct position of the catheter during surfactant administration
Drugs and non-pharmacological analgesic interventions
Knowledge about the indications, dosage, correct administration, effects, and side effects of drugs that may be used as part of the LISA procedure
Knowledge about advantages and disadvantages of using sedation and analgesia as part of the LISA procedure
Knowledge about use of non-pharmacological analgesic interventions as part of the LISA procedure
Airway management
Practical skills in optimizing positioning of the patient including manual airway maneuvers (e.g., jaw lift)
Knowledge about the neonatal upper respiratory and lower respiratory airway anatomy
Knowledge about airway visualization techniques using a laryngoscope
Knowledge about indications for intubation in terms of failure criteria for the LISA procedure
Practical skills in endotracheal intubation
Respiratory support
Practical skills in mask ventilation using a bag-valve-mask or T-piece
Practical skills in non-invasive ventilation
Knowledge of indications for CPAP
Practical skills in using nasal CPAP
Physical examinations and vital signs
Knowledge about target vital sign values such as heart rate and saturation
Knowledge about how to recognize signs of pain and distress
Evaluation and outcomes
Knowledge about how to evaluate LISA procedure success or failure
Other skills
Knowledge about other methods of surfactant administration
Practical skills in treatment of pneumothorax
Knowledge about chest X-ray interpretation in newborn infants with signs of respiratory distress
Knowledge about blood gas interpretation
Knowledge about maintaining thermal control during the LISA procedure
Practical skills in neonatal life support

The approved LISA-CUR.

*CPAP* continuous positive airway pressure, *LISA* less invasive surfactant administration, *LISA-CUR* LISA curriculum, *LISA-AT* LISA assessment tool, *RDS* respiratory distress syndrome.

**Table 4:** The final approved LISA assessment tool (LISA-AT) with response anchors one, three, and five after *Delphi round 3*.

Pre-procedure	1	2	3	4	5
Monitoring *Ensure appropriate monitoring of vital signs before and during the LISA procedure.*	Fails to ensure monitoring.		Competent but not consistent monitoring of vital signs.		Excellent monitoring of vital signs before and during the procedure.
Equipment preparation *Ensure all equipment used in the LISA procedure is functioning.*	Fails to check equipment/checks equipment incorrectly.		Competent but not consistent check of the equipment.		Excellent check of all the equipment.
Positioning *Position the patient optimally and maintain position during the LISA procedure*.	Fails to position the patient.		Competent but not consistent position of the patient throughout the procedure.		Excellent position of the patient during the procedure.
Team-briefing/Team resource management *Perform a team briefing before the LISA procedure including task assignment, outlining potential problems, etc*.	Fails to perform team briefing.		Performs team briefing with some experience.		Excellent team briefing.

Procedure	1	2	3	4	5
Pharmacological interventions *Check drugs and doses that may be used as part of the LISA procedure (including antidote if opioid is being used)*.	Fails to check drugs.		Competent but not consistent check of drugs.		Excellent check of all drugs and doses.
Non-pharmacological interventions *Apply non-pharmacological techniques for pain and stress management*.	Poor application of non-pharmacological techniques.		Some application of non-pharmacological techniques		Full application of non-pharmacological techniques.
Laryngoscopy *Competent and non-injurious handling of the laryngoscope and good visualization of the vocal cords including sufficient overview of the airway*.	Fails to handle the laryngoscope/injurious handling of the laryngoscope/fails to visualize the vocal cords.		Competent but not consistent handling of the laryngoscope.		Excellent handling of the laryngoscope.
Catheterization *Insert the catheter into the trachea at the desired depth and maintain the position during instillation while retracting the laryngoscope*.	Fails to insert the catheter.		Competent but not consistent handling of the catheter.		Excellent handling of the catheter.
Surfactant administration *Slow infusion to allow surfactant to be inhaled by the infant*.	Fails to administer surfactant correctly.		Competent but not consistent surfactant administration.		Excellent surfactant administration.
Complications *Manage possible complications during administration*.	Fails to manage complications.		Manages complications with some experience.		Excellent management of complications.
Respiratory support *Maintain CPAP/NIV during the LISA procedure*.	Fails to maintain CPAP/NIV during the procedure.		Competent but not consistent use of CPAP/NIV during the procedure.		Excellent use of CPAP/NIV during the procedure.

Non-technical skills	1	2	3	4	5
Non-technical skills *Situational awareness, communication, teamwork, team leadership skills*.	Fails to use non-technical skills.		Use non-technical skills with some experience.		Excellent use of non-technical skills.

Overall	1	2	3	4	5
Number of attempts *Number of attempts until the LISA procedure is successfully performed*.	Procedure successfully performed only after more than 3 attempts, or procedure is abandoned.		Procedure successfully performed with 2 or 3 attempts.		Procedure successfully performed at the first attempt.
Adherence to algorithm / Time factor *Able to follow this algorithm and minimize time delay in treatment*.	Fails to follow the algorithm.		Able to follow the algorithm but with significant time delay.		Able to follow the algorithm with minimal time delay.
Handling of the infant *Gentle handling of the patient during the LISA procedure*.	Fails to handle the patient in a gentle way.		Handles the patient with some experience.		Excellent handling of the patient.
Total item score (15–75): ______					
Overall assessment:					
*() PASS: Competent, safe, and gentle performance of the LISA procedure*					
*() BORDERLINE: Partially competent with adherence to safety requirements, but more training is needed*					
*() FAIL: Competence and safety not demonstrated*					

The approved LISA-AT consists of 15 items, each rated on a 5-point Likert scale. The minimum score is 15 points, and maximum score is 75 points. The LISA-AT obtained 98% approval among the participating LISA experts.

*CPAP* continuous positive airway pressure, *LISA* less invasive surfactant administration, *LISA-CUR* LISA curriculum, *LISA-AT* LISA assessment tool, *NIV* non-invasive ventilation.

### Delphi round 1

The experts suggested 807 and 658 items for the LISA-CUR and the LISA-AT, respectively.

For the LISA-CUR, 148 and 498 of the 807 items were excluded being duplicates or according to the following criteria: teaching activities, learning-teaching strategies, and teaching methods. The remaining 159 unique items were further condensed to 44 items and post hoc organized into the following nine categories to achieve a better overview: (1) *Indications and contraindications*, (2) *Complications*, (3) *Familiarity with the equipment*, (4) *Drugs and non-pharmacological measures*, (5) *Airway management*, (6) *Respiratory support*, (7) *Monitoring and assessment*, (8) *Evaluation*, (9) *Other skills* (Online Supplement, Appendix B).

For the LISA-AT, 256 and 314 of the 658 items were excluded for the same reasons as those listed for the LISA-CUR. The steering committee further condensed the remaining 88 unique items to 22 and post hoc sorted all items chronologically in descending order from procedure start to finish (Online Supplement, Appendix C). These two lists were used in *Delphi round 2* and returned to the experts for assessment.

The list of training equipment used to practice LISA is available in the Online Supplement, Appendix D.

### Delphi round 2

The response rate was 82% (126/153 experts).

For the LISA-CUR, 12 items were eliminated by consensus (consensus score <80%, Online Supplement, Appendix B). Between rounds 2 and 3, the steering committee decided to merge three of the included items based on the experts’ comments resulting in a preliminary LISA-CUR of 29 items.

For the LISA-AT, eight items were eliminated by consensus (Online Supplement, Appendix C), resulting in a preliminary LISA-AT of 14 items. One item, “*Surfactant administration synchronized with the patient’s inspiration with a closed mouth*”, was rephrased by the steering committee to “*Surfactant administration: Slow infusion to allow surfactant to be inhaled by the infant*” based on the comments received. Round 2 was repeated for this item alone.

The steering committee assigned specific response anchors on a five-point Likert scale to each item included in the LISA-AT based on the experts’ suggestions. The steering committee provided precise guidance on the “poor,” the “sufficient,” and the “excellent” performance (response anchors 1, 3, and 5, respectively) for each item to hopefully increase inter-rater reliability and provide more standardized assessments without restricting the use of the LISA-AT, as operators may perform very differently.

The complete overview of adjustments to the LISA-CUR and LISA-AT between rounds 2 and 3 is available in the Online Supplement, Appendices E and F, respectively.

### Delphi round 3

The response rate in *Delphi round 3* was 83% (127/153 experts). The LISA-CUR and LISA-AT achieved consensus without changes (100% and 98%, respectively). Furthermore, the rephrased item was included in the final assessment tool as 86% of the experts rated the item 4 (important) or 5 (very important).

## Discussion

In this Delphi process, 153 LISA experts from 14 countries were involved in a three-round iterative process to gather consensus regarding the training and assessment of LISA operators. Together, we developed the LISA-CUR consisting of 29 unique items, and the LISA-AT consisting of 15 unique items with response anchors. There are several benefits of implementing the LISA-CUR and LISA-AT in standardized training after adaptation to the local setting to increase operator competence and patient safety.

Until recently, a lack of formalized training^18^ has been a barrier to the implementation of LISA, as confirmed by experts’ in our study. Furthermore, the majority of the LISA experts in our study only performed 0–5 LISA procedures per month. Lack of formal training and infrequent clinical exposure to the LISA procedure may challenge adherence to clinical guidelines.^15,26^

A combination of theoretical aspects with practical training for learning the LISA procedure is recommended.^14^ So far, a consensus on a LISA training curriculum or assessment tool to evaluate operator competence has yet to be published. In 2021, Liebers et al.^17^ developed a LISA training program including multimedia materials, checklists, pocket cards, and team briefing, which increased the mean success rate of LISA from 62% to 92%, defined as no need for any of the following: intubation within the following 72 h, a second surfactant dose, or termination of the LISA procedure. In 2019, Vento et al.^15^ published a practical recommendation for surfactant administration specifying the characteristics of a trainer and a trainee and how training should be structured. Three training modules for operators and assistants were proposed, including (1) pre-course preparation and online test, (2) LISA training on a mannequin, and (3) post-course LISA consolidation by performing several procedures in the clinical setting with supervision combined with periodic refreshers on a mannequin. Recommendations by Vento et al.^15^ and Reynolds et al.^27^ suggest treatment thresholds, exclusion criteria, personnel, monitoring, equipment, non-pharmacological measures, and analgesics, including doses. The LISA-CUR may work with these existing recommendations to optimize and standardize LISA training in the future.

The consensus-based LISA-AT consists of 15 unique items and may provide standardized feedback, assessment, and potential certification of LISA operators. Vento et al. (2019)^15^ and Reynolds et al. (2021)^27^ also identified core items that an operator should be able to undertake, but these reflected the individual opinions of the clinicians involved in formulating the recommendations. All their core items were included in the LISA-AT we propose and are now supported by international expert consensus. Various techniques for the LISA procedure exist,^15^ which may explain the lack of consensus achieved for various procedure-specific items like catheter type, Magill forceps, checking for gastric retention, and lung ultrasound, which were excluded from the final LISA-CUR and LISA-AT. These procedural factors clearly should be a focus of future research. Local protocols for each procedure step must exist before the LISA-CUR and LISA-AT can be adapted to the local setting and successfully implemented. Since this is first of a kind initiative, opinions may be inconsistent and further validation of the tool will help to refine it.

Development of the LISA-AT is the first step to enable mastery learning through standardized, continuous feedback and assessment until a predefined performance criterion is achieved.

However, there is currently no validity evidence to support the use of the LISA-AT and its ability to differentiate between operators with different levels of experience.^21^ To our knowledge, there is no pre-defined mastery learning level, and we do not know how much practice is needed during simulation-based LISA training to reach sufficient levels of mastery.^9,28^ Future studies need to validate the LISA-AT using Messick’s framework,^29^ which is the gold standard when evaluating validity as the American Educational Research Association recommended in their Standards for Education and Psychological Testing.^30,31^ Following validation, NICUs and neonatal simulation centers may standardize LISA training and assessment by implementing the LISA-CUR and LISA-AT. This should be a necessary step before the operator is promoted from training in the simulated environment to training in the clinical setting.

The Delphi research method is ideal for studying which items to include in the LISA-CUR and the LISA-AT when objective information is unavailable.^32^ We minimized the risk of conflict of interest and group pressure frequently associated with expert panels by anonymizing all responses prior to analyses and providing a unique link for each expert per round.^32,33^ This study was conducted following a detailed protocol (available from NB upon request) with no changes to the study design after the initiation of round 1, limiting individual members’ influence through all three rounds. Hence, only items achieving the predefined consensus cut-off were included in the final LISA-CUR and LISA-AT. Due to the online advertisement through the ESPR website, we are unaware of the total number of potential experts who might have seen the invitation. We are also unaware of the actual number of participants recruited by the online advertisement and the reasons for non-participation at each stage. However, we minimized the risk of bias from lack of response by achieving response rates of more than 80% of the initial responders in Delphi rounds two and three. Multiple NICUs contributed data from each country, minimizing the risk of bias arising from multiple responses from a single institution. No single country was represented by more than 15% of all the experts in our study. We chose a broad set of inclusion criteria for LISA experts to gather as many inputs as possible. Although this might have diluted the qualification of being an “expert,” baseline data showed that 97% of experts were board-certified neonatologists involved with the LISA procedure for several years. The experts involved in this study represented highly specialized NICUs from Western and non-Western countries. However, some NICUs may not have the resources to improve training and assessment for the LISA procedure, as this procedure is just one part of a resource-demanding intensive care strategy for preterm infants.

## Conclusion

This Delphi process generated international consensus on a LISA curriculum (LISA-CUR) and a LISA assessment tool (LISA-AT). The LISA-CUR and LISA-AT are published with this study and may be used by LISA trainers, supervisors, and curriculum developers to guide their efforts.

## Supplementary information

APPENDIX A

APPENDIX A

APPENDIX B

APPENDIX C

APPENDIX D

APPENDIX E

## Data Availability

The datasets generated during and/or analyzed during the current study are available from the corresponding author on reasonable request.
