# Supplementary material for: Curriculum and assessment tool for less invasive surfactant administration: an international Delphi consensus study
Source: Pediatr Res. 2023 May 4;94(3):1216–24. doi: 10.1038/s41390-023-02621-2 (PMC10444608; doi:10.1038/s41390-023-02621-2)
Supplement: Supplementary file 4 — APPENDIX C [file 41390_2023_2621_MOESM4_ESM.pdf]

APPENDIX C: List of training equipment in LISA

1. Manikin (newborn/premature)
2. Catheter and/or nasogastric tubes
3. Regular/video laryngoscope depending on local practice
4. Standard airway management kit including a resuscitation bag
5. Non-invasive ventilation equipment (e.g., nCPAP/NIPPV device)
6. CO_2 detector
7. Suctioning catheter
8. Magill’s forceps depending on local practice
9. IV catheters
10. Blankets
11. Syringes in 5 and 10 ml
12. Lung ultrasound with linear probe depending on local practice
